# Towards earlier diagnosis and treatment of disorders of the brain

**DOI:** 10.2471/BLT.17.206599

**Published:** 2018-05-01

**Authors:** Monica Di Luca, David Nutt, Wolfgang Oertel, Patrice Boyer, Joke Jaarsma, Frederic Destrebecq, Giovanni Esposito, Vinciane Quoidbach

**Affiliations:** a Department of Pharmacological and Biomolecular Sciences, University of Milan, Via Balzaretti 9, 20133 Milano, Italy.; b European Brain Council (EBC), Brussels, Belgium.

The 2015 Global Burden of Disease study estimates that about a third of the population worldwide is affected by mental or neurological disorders across their lifespans.^1^ This high burden^2^^,^^3^ may be surprising as there is a general lack of awareness on the pervasiveness of disorders of the brain. Global data, but particularly those from European studies, indicate that these disorders are a major public health problem: disorders of the brain rank among the leading causes of ill-health and disability and account for 35% of Europe’s total disease burden with a yearly cost of 800 billion euros, of which 60% are related to direct health care and non-medical costs.^4^^,^^5^ The burden is growing due to the epidemiological transition from acute to chronic diseases and the increase in life expectancy, but also because of several socioeconomic, environmental and behavioural health determinants.

Mental and neurological disorders are complex and are linked to hundreds of specific diagnoses.^6^^,^^7^ The causes of such disorders are heterogeneous, ranging from pathological protein aggregation leading to neurodegeneration or dysregulation of the immune process, to developmental and functional abnormalities. These disorders also frequently involve an intricate interplay between genetic and environmental factors. Needs for basic and clinical research, the provision of medicines and medical devices, and adequate health-care systems and services are growing, but are increasingly unmet.

Discussions on health care focus too often on the increase of health-care cost rather than on the benefits of better health. Therefore, emphasizing on the need for more value-based and patient-centred care, and for the scaling-up of an integrated care model for mental and neurological disorders is important. An integrated care model encompasses the whole care process, from prodromal, early diagnosis to disease management and patient empowerment.^8^

The European Brain Council, an organization promoting research in Europe on health and disorders of the brain to improve the quality of life of those living with such disorders, initiated a two-year research project on the value of treatment.. The project included case studies on schizophrenia, Alzheimer disease, epilepsy, headache, normal pressure hydrocephalus, Parkinson disease, multiple sclerosis, restless legs syndrome and stroke. The study’s research framework included the testing of an integrated model and the development of a series of qualitative and quantitative benchmarks to identify treatment gaps and causal factors along the continuum of care in a patient care pathway analysis. The study also estimated the socioeconomic impact and health gains from best practice health-care interventions with an economic evaluation. Case studies were analysed in collaboration with hundreds of experts from the European Brain Council in line with the research framework, applying empirical evidence from different European countries.

In June 2017, the council published the outcome of these case studies in *The value of treatment for brain disorders – policy white paper.*^9^ The publication provides important new insights into recent progress in the areas of pharmacology and the biopsychosocial approach, and into health-care service delivery and integrated care for brain disorders. Conclusions link early detection and diagnosis of disease, as well as timely intervention, to measurable health gains such as improved survival rate, reduced complications and disability, better quality of life and lower treatment costs. Primary prevention; modification of lifestyle factors and control of vascular risk factors, effective therapy in the prodromal stages, and secondary prevention; including diagnostic biomarkers and routine mental health screening, remain essential.

For instance, the treatment success rate in schizophrenia can be improved if patients at risk are identified, psychotic symptoms are detected early and treatment is initiated in the prodromal phase. Depending on the stage of the disorder, antipsychotic medication, psychosocial interventions or both, are needed.^10^ In multiple sclerosis, the key paradigm is early diagnosis and use of disease-modifying treatment. Such treatment at the early stage of multiple sclerosis can slow disease progression and subsequent disability. Primary and secondary prevention of modifiable risk factors can avert long-term disability due to multiple sclerosis and reduce its economic consequences.^11^^,^^12^ However, many mental and neurological disorders still lack a cure and more studies are needed to understand the causes and reasons for progression of each disease. Research is also needed to develop new treatments that modify disease progression in addition to improving symptoms.
